# Layperson's View

**DOI:** 10.1371/journal.pmed.0020363

**Published:** 2005-10-25

**Authors:** Simone Autry

**Affiliations:** **1**New York, New YorkUnited States of America

In regards to the media's dissemination of health information [1], I think there are the following three problems. Firstly, the media are shamefully sensational. Headlines can be misleading just to draw attention to them. Secondly, the media publish conflicting study reports (often going back and forth between conflicting findings more than once). Then, they find people to dispense advice based on these reports (also going back and forth multiple times) without giving good explanations as to why there are conflicting reports. Thirdly, if I were to summarize my thoughts about what I hear in the media, it would be that everything fights cancer if it doesn't kill you first. What was good for you last week will kill you this week. There is no point, then, in following advice.
